# Regulation of Reactive Oxygen Species and the Antioxidant Protein DJ-1 in Mastocytosis

**DOI:** 10.1371/journal.pone.0162831

**Published:** 2016-09-09

**Authors:** Do-Kyun Kim, Michael A. Beaven, Joseph M. Kulinski, Avanti Desai, Geethani Bandara, Yun Bai, Calman Prussin, Lawrence B. Schwartz, Hirsh Komarow, Dean D. Metcalfe, Ana Olivera

**Affiliations:** 1 Mast Cell Biology Section, Laboratory of Allergic Diseases, National Institute of Allergy and Infectious Diseases, National Institutes of Health, Bethesda, Maryland, United States of America; 2 Laboratory of Molecular Immunology, National Heart, Lung and Blood Institute, National Institutes of Health, Bethesda, Maryland, United States of America; 3 Department of Internal Medicine, Virginia Commonwealth University, Richmond, Virginia, United States of America; Toho Daigaku, JAPAN

## Abstract

Neoplastic accumulation of mast cells in systemic mastocytosis (SM) associates with activating mutations in the receptor tyrosine kinase KIT. Constitutive activation of tyrosine kinase oncogenes has been linked to imbalances in oxidant/antioxidant mechanisms in other myeloproliferative disorders. However, the impact of *KIT* mutations on the redox status in SM and the potential therapeutic implications are not well understood. Here, we examined the regulation of reactive oxygen species (ROS) and of the antioxidant protein DJ-1 (PARK-7), which increases with cancer progression and acts to lessen oxidative damage to malignant cells, in relationship with SM severity. ROS levels were increased in both indolent (ISM) and aggressive variants of the disease (ASM). However, while DJ-1 levels were reduced in ISM with lower mast cell burden, they rose in ISM with higher mast cell burden and were significantly elevated in patients with ASM. Studies on mast cell lines revealed that activating *KIT* mutations induced constant ROS production and consequent DJ-1 oxidation and degradation that could explain the reduced levels of DJ-1 in the ISM population, while IL-6, a cytokine that increases with disease severity, caused a counteracting transcriptional induction of DJ-1 which would protect malignant mast cells from oxidative damage. A mouse model of mastocytosis recapitulated the biphasic changes in DJ-1 and the escalating IL-6, ROS and DJ-1 levels as mast cells accumulate, findings which were reversed with anti-IL-6 receptor blocking antibody. Our findings provide evidence of increased ROS and a biphasic regulation of the antioxidant DJ-1 in variants of SM and implicate IL-6 in DJ-1 induction and expansion of mast cells with *KIT* mutations. We propose consideration of IL-6 blockade as a potential adjunctive therapy in the treatment of patients with advanced mastocytosis, as it would reduce DJ-1 levels making mutation-positive mast cells vulnerable to oxidative damage.

## Introduction

Reactive oxygen species (ROS) are formed in response to receptor tyrosine kinase stimulation and have important functions in cell signaling and cellular processes. Abnormally increased levels of ROS are observed in hematopoietic malignancies [1], although their role in cancer pathology requires clarification due to the involvement of ROS in cellular functions that may be beneficial or harmful depending on the context of the disease [2–4]. Imbalances between ROS and antioxidant molecules, however, do result in oxidative stress. Oxidative stress and altered redox status is characteristic of malignant cells which become more dependent on antioxidant mechanisms for survival as they transform, and this feature is viewed as a vulnerability that can be exploited when considering treatment strategies [4, 5]. Among antioxidant proteins, DJ-1 (or PARK7) is evolutionary conserved and confers cell protection against oxidative damage. DJ-1 was originally described as an oncogene product [6] and its levels are elevated in a number of malignancies in correlation with poor prognosis [7–9]. The oncogenic activity of DJ-1 in part appears to relate to its ability to increase a cell's resistance to ROS [10, 11]. DJ-1 thus acts as a scavenger of ROS by undergoing oxidation during which it is degraded [7, 12–14], and by directly activating [15] or inducing transcription of other antioxidant enzymes [7, 16]. However, little is known about DJ-1 levels in association with ROS and the factors that regulate them in hematopoietic malignancies.

Mastocytosis is a myeloproliferative disorder characterized by the accumulation of neoplastic mast cells within tissues [17]. Systemic mastocytosis (SM) is frequently associated with gain-of-function mutations in codon 816 (D816V) of KIT, the tyrosine kinase receptor for stem cell factor (SCF) [17–19]. An elevation of oxidized protein products has been reported in mastocytosis of the skin and in indolent SM (ISM) [20], although the cause for this occurrence and whether it correlated with actual increases in ROS levels was not investigated. Furthermore, it is not known whether progressive pathology in SM associates with rising ROS levels. SM includes variants with increasingly severe disease, being the numbers of neoplastic mast cells, along with serum tryptase levels and serum IL-6 levels, normally highest in patients with the most extensive disease and poor prognosis [19, 21–23]. Previous reports demonstrated that ROS are generated during proliferation and/or activation of cultured mast cells [24–26]. In addition, antigen-mediated ROS accumulation is enhanced in DJ-1-null mast cells, and activation of dermal mast cells causes increases in serum ROS, particularly in DJ-1 deficient mice [27]. However, whether activation of KIT and the mutational status of *KIT* can regulate ROS and DJ-1 in mast cells is unknown.

Because DJ-1 is linked to mast cell activity, oxidative regulation and cancer progression, we thus investigated whether DJ-1 is dysregulated in mastocytosis in association with disease severity and in a model of mastocytosis, as well as the possible impact of *KIT* mutations on DJ-1 and ROS levels. We found that in less severe disease, the levels of the antioxidant DJ-1 are reduced while, in contrast, they are increased in patients with high mast cell burden and advanced disease. Based on the models of disease used here, it appears that the reduction in DJ-1 in less advanced disease relates to *KIT* mutations causing increased ROS production coupled to DJ-1 degradation, while in more aggressive disease, IL-6 mediates DJ-1 transcription and counterbalances raising oxidative stress and favors mast cell growth. This study defines previously unrecognized factors regulating the levels of ROS and DJ-1 in SM in relation to mast cell burden and identifies DJ-1 as well as IL-6 and its signals as possible targets for therapeutic intervention.

## Materials and Methods

### Patient population

Human serum samples were obtained after informed written consent in accordance with protocols 02-I-0277 and 09-I-0049 approved by the National Institute of Allergy and Infectious Diseases Institutional Review Board. Some samples were obtained in collaboration with Dr. Lawrence Schwartz (Virginia Commonwealth University, VCU). These samples were residual de-identified clinical serum samples that had been submitted to Dr. Schwartz’s Clinical Diagnostic Immunology Lab for monitoring tryptase levels in his patients with advanced mastocytosis, and had been stored at -75°C. The VCU IRB (HM20007397) waived informed consent for research use of these samples in the current study. Serum samples were obtained from 85 adult patients with ISM, SSM or ASM classified according to a World Health Organization (WHO) consensus classification [28–30] and 28 adult healthy controls. Patients and healthy volunteers had a similar median age of 45 years and 48 years, respectively. Serum samples were blinded before analysis.

### Mouse model of mastocytosis

These studies were performed in accordance with NIH guidelines and with the animal study proposal (LAD2E) approved by the NIH/NIAID Animal Care and Use Committee. Female DBA/2 mice (8–10 weeks) from Charles River Laboratories (Boston, MA) were injected i.v. with 10^2^ to 2 x 10^2^ P815 murine mastocytoma cells [31] (up to 4 passages) grown as specified by ATCC (ATCC, Manassas, VA) or bone marrow mast cells (BMMC) cultured as described from DBA/2 mice [27]. Cells were washed and injected into mice in 200 μl of RPMI. Serum DJ-1, ROS and IL-6 were measured at the indicated times. On day 10 after transfer of P815 cells, mice were injected daily (i.p.) with 200 μg of anti-IL-6 receptor antibody in 200 μl of PBS (Clone MP5-20F3; R&D systems, Minneapolis, MN), an isotype control (R&D systems, Minneapolis, MN) or PBS. After euthanasia on day 16 by CO_2_ inhalation, tissues were placed in buffered formalin, paraffin-embedded and stained with alcian blue/safranin. Blood collected with heparinized syringes was used for FACS analysis.

### Flow cytometry

Heparinized blood samples were treated with ACK lysis buffer (Quality Biological Inc., Gaithersburg, MD) to lyse red blood cells, washed and resuspended in FACS buffer (PBS, 2% FCS, 0.05% sodium azide) at 1 x 10^7^ nucleated cells/ml. Cells were pre-stained with an amine-reactive aqua live/dead dye (Life Technologies, Waltham, MA) followed by Fc block (clone 24G2), then incubated with anti-CD117 (ACK2) allophycocyanin (APC) and anti-FcεRI (Mar-1) r-phycoerythrin (PE) antibody conjugates (eBioscience, San Diego, CA). Data acquisition was performed on a LSR II flow cytometer (BD Biosciences, Sparks, MD) and analyzed using FlowJo software (Tree Star, Ashland, OR).

### Mast cell line cultures and cell activation

Human HMC-1 mast cell lines (HMC-1.1 with the G560V mutation and HMC-1.2 with the G560V and D816V *KIT* mutations) were a kind gift from Dr. JH Butterfield (Mayo Clinic, Rochester, MN) and were obtained from a patient with mast cell leukemia accepted in the Mayo Clinic in 1998 after written informed consent [32]. LAD2 (expressing normal KIT) mast cells were previously derived from bone marrow biopsies of a patient under an NIAID protocol after written informed consent for use in research and for publishing and distributing the cell line [33]. HMC-1 and LAD2 cell lines were cultured as described [32, 33]. HMC-1 cells were stimulated with 100 ng/ml SCF or 50 ng/ml IL-6 for different time periods. LAD2 cells were plated in culture media without SCF for 24 h and then stimulated with 100 ng/ml SCF for the indicated times. For inhibition of the proteasome, cells were treated with 10 μM of the proteosomal inhibitor MG132 for 6 h. Tocilizumab, a neutralizing anti-IL-6 receptor (anti-IL-6R) antibody, and inhibitors of JAK (TG101348, 1 μM) (Selleck Chemicals; Houston, TX) or STAT3 (NSC74859, 100 μM) (EMD Millipore) were added 2 h (in LAD2 cells) or 14 h (in HMC-1.2 cells) before IL-6 stimulation. In some experiments, LAD2 cells were treated with SCF for 48 h and then stimulated with IL-6 (50 ng/ml), IL-31 (50 ng/ml; R&D Systems, Minneapolis, MN) or histamine (1 to 50 μM; Sigma-Aldrich, St Louis, MO).

MCBS1 cells [34], which lack surface KIT expression, were stably transfected with pMX-puro/KIT WT or D816V-KIT as described previously [35]. MCBS1 cells were cultured in media containing 3 μg/ml puromycin as described. Cells were placed in puromycin-free culture media for 24 h and then plated in 12 well plates (2 x 10^5^ cells/ well; 1 ml) for additional 24 h. Media were collected for measurement of ROS and cells lysates for DJ-1 determinations. Expression of KIT and KIT phosphorylation in the transfected cells was confirmed by western blot and presence of mutation in the obtained cell line was confirmed by allele specific PCR [36].

### Tryptase, DJ-1, ROS and IL-6 measurements

Tryptase levels were determined by immunoassay using the UNICAP system (Pharmacia-Upjohn, Peapack, NJ) [22]. Measurements of IL-6 levels and extracellular DJ-1 were performed by ELISA (R&D Systems, Inc., Minneapolis, MN) while intracellular DJ-1 was detected by immunobloting. Measurements of DJ-1 levels in sera and released from cells were performed using a DuoSet ELISA (R&D Systems) where 96-well ELISA plates (BD Falcon; San Jose, CA) were coated with goat anti-human DJ-1 (0.4 μg/ml in PSB) and the immune assay performed following manufacturer’s specifications. DJ-1 appeared to be labile to freeze-thaw cycles, particularly in the sera of patients with more aggressive disease. However, assay controls indicated full recovery of recombinant DJ-1 when added exogenously to serum samples from different individuals indicating no differences in the stability of DJ-1 in these samples. For determination of DJ-1 in mouse sera, a mouse DJ-1 specific ELISA (American Research Products, Inc., Walthham, MA) was used following manufacture’s specifications.

Intracellular ROS levels were measured with the OxiSelect^™^ Intracellular ROS Assay kit (Cell Biolabs, Inc., San Diego, CA). LAD2 (2×10^5^ cells/1.5 ml) and HMC-1 (2×10^5^ cells/1.5 ml) cells were incubated with 2’, 7’-dichlorofluorescin diacetate (DCFH-DA) (20 μM) for the last 20 min of stimulation with SCF or IL-6 at 37°C and then washed in cold-PBS. DCFH-DA is diffused into cells and deacetylated by cellular esterases to non-fluorescent 2’, 7’-Dichlorodihydrofluorescin (DCFH). Oxidation of DCFH by ROS and/or reactive nitrogen species (RNS) results in a fluorescent derivative 2’,7’-dichlorofluorescein (DCF) whose fluorescence is proportional to the total ROS/RNS levels in the sample. Cells were lysed in 100 μl of 0.5% Triton-X 100 and their fluorescence was monitored at 492 nm excitation/535 nm emission using a Wallac Victor fluorescent plate reader (Perkin Elmer, Shelton, CT). ROS/RNS levels were calculated by interpolation into a H_2_O_2_ standard curve.

Similarly, total free radicals in the culture medium or in serum samples were measured using the OxiSelect *In Vitro* ROS/RNS Assay Kit (Cell Biolabs, Inc.) that uses stabilized DCFH instead of DCFH-DA, which is for use in intact cells. The stabilized, highly reactive DCFH form is oxidized by ROS in the samples in the presence of a catalyst provided by the manufacturer to help accelerate the reaction. Serum samples were diluted in PBS (1:100), and 50 μl of the diluted samples were assayed following manufacturer’s protocol. Fluorescence of the oxidized DCFH to DCF is proportional to the concentration of ROS in the samples and was measured as above. Previous studies have shown that stimulated mouse and human derived mast cells produce ROS but not nitric oxide [37]. For this reason DCF fluorescence in these assays have been attributed to ROS.

### Quantitative real-time PCR

Total cellular RNA was extracted from 5×10^5^ cells (LAD2, HMC-1.1 and HMC-1.2) using an RNeasy Plus Mini kit (Qiagen, Valencia, CA). A 1 μg aliquot of RNA, as determined by spectrophotometry, was converted to cDNA by reverse transcription using random hexamers and SuperScript III reverse transcriptase (Invitrogen Life Technologies, Carlsbad, CA) in a 20 μl reaction mixture according to the manufacturer’s instructions. Quantitative PCR was executed in 96-well plates using ABI Prism 7500 Real Time PCR system (Foster City, CA) and TaqMan gene expression assays for DJ-1 (AIVI5HL) and GAPDH as the endogenous control gene (Assay ID: Hs99999905_m1) (Life Technologies, Grand Island, NY) was used for normalization.

### Knockdown of DJ-1 by sh-RNA

DJ-1-targeted short hairpin RNAs (sh-RNAs) were purchased from Sigma-Aldrich (Sigma-Aldrich, St. Louis, MO). The MISSION^®^ shRNA constructs were as follows: SHC002, nontarget control vector, and TRCN0000004921 as sh-RNA for DJ-1. The latter was selected among other constructs tested in LAD2 cells (TRCN0000004918, TRCN0000004919 and TRCN0000004920) as the one producing most consistent silencing effects, although all worked similarly. The Mission Lentiviral packaging mix (Sigma-Aldrich) and the pLKO1 transfer vectors with sh-DJ-1 or control sh-RNA (3.4 μg) were cotransfected into 293T cells (4 × 10^6^ cells) with FuGENE6 transfection reagent (Roche, Indianapolis, Ind) in Opti-MEM as described. [38]. The media were removed and replaced with fresh media 19 h after transfection, and the lentivirus was collected by centrifugation (25,000 rpm for 1 hour 40 minutes at 4°C) 62 to 65 hours after transfection. The resulting pellet was resuspended in 3 mL of prewarmed complete StemPro medium (Invitrogen, Carlsbad, CA) and 0.5 to 1.0 ml of this volume transferred to a T25 culture flask containing 1 to 2 × 10^6^ HMC-1.2 in 10 mL of complete StemPro medium. Two days after transduction, cells were placed in fresh complete media for two additional days. Transduced cells were selected in media containing 0.2 μg/mL puromycin (Sigma-Aldrich) for 5 days. After a recovery period of 2 days, the cells were used for experiment.

### Statistical analysis

ONE-way ANOVA was used to determine statistically significant differences between groups using SigmaStat software (Point Richmond, CA). Data on human samples were analyzed using unpaired, non-parametric, Mann-Whitney test. Correlations between parameters were analyzed with the Spearman correlation coefficient using SigmaStat software. P values <0.05 were considered significant.

## Results

### Serum levels of ROS and DJ-1 in patients with mastocytosis

**Fig 1:**
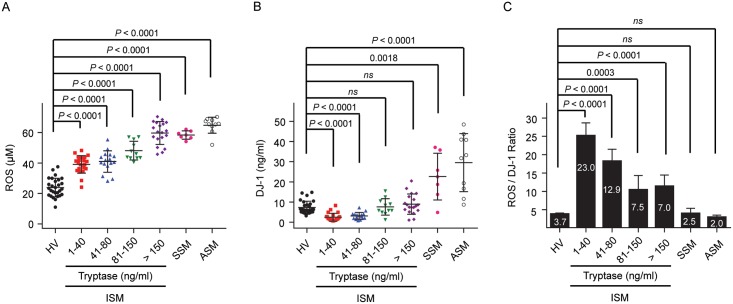
Alterations in ROS and DJ-1 in mastocytosis patients. Serum levels of ROS (A) and DJ-1 (B) in healthy adult volunteers and in adult patients with ISM, divided into groups according to their tryptase serum levels (1–40 ng/ml, n = 22; 41–80 ng/ml, n = 16; 81–150 ng/ml, n = 11; > 150 ng/ml, n = 19), SSM (n = 7) and ASM (n = 10). Serum levels of ROS and DJ-1 were measured by a fluorescent assay and by ELISA, respectively. Horizontal bars represent the median. P values of the different comparisons are indicated. (C) Ratio of ROS to DJ-1 levels for each patient sorted according to their serum tryptase levels or disease variant. Values are the median±SEM.

We measured the levels of ROS and DJ-1 in the sera from 85 patients with SM, including ISM, the most common and the least severe variant of SM, smoldering systemic mastocytosis (SSM) which is a variant that shows indications of slow progression and over time results in a high burden of neoplastic cells [28], and aggressive SM (ASM), a variant that can be life threatening [28–30]. The ISM population was grouped according to serum tryptase levels, which have been used as a measure of mast cell burden in this disease [21, 22, 39]. Serum levels of ROS were elevated in all patients with SM, particularly in those with ISM with higher tryptase values (>80 ng/ml) and in patients with more aggressive mastocytosis (SSM and ASM) (Fig 1A). DJ-1 levels showed a biphasic regulation in that they were diminished in ISM with tryptase below 80 ng/ml, tended towards normal values in patients with tryptase levels >80 ng/ml and were elevated in patients with smoldering or aggressive disease (Fig 1B). This biphasic regulation was reflected in a markedly higher than normal ratio ROS/DJ-1 in ISM with serum tryptase values < 80 ng/ml and a decline in this ratio towards normal values in patients with ISM with tryptase values >80 ng/ml, whereas the ratios in SSM and ASM groups were not significantly different than that of the healthy volunteers (Fig 1C). These changes in ratio imply that the mechanisms for regulating levels of ROS and DJ-1 are more complex than a simple direct relationship to mast cell accumulation and suggest that the underlying mechanisms change as mast cell burden increases. While diminished levels of the antioxidant DJ-1 are consistent with the increased ROS in ISM with lower tryptase values, the substantially elevated DJ-1 in association with more aggressive disease requires explanation, and the consequences of which would be to lessen ROS levels as disease progresses and thus promote survival of malignant cells [11].

### Mastocytosis cell lines show increased ROS and reduced levels of DJ-1

**Fig 2:**
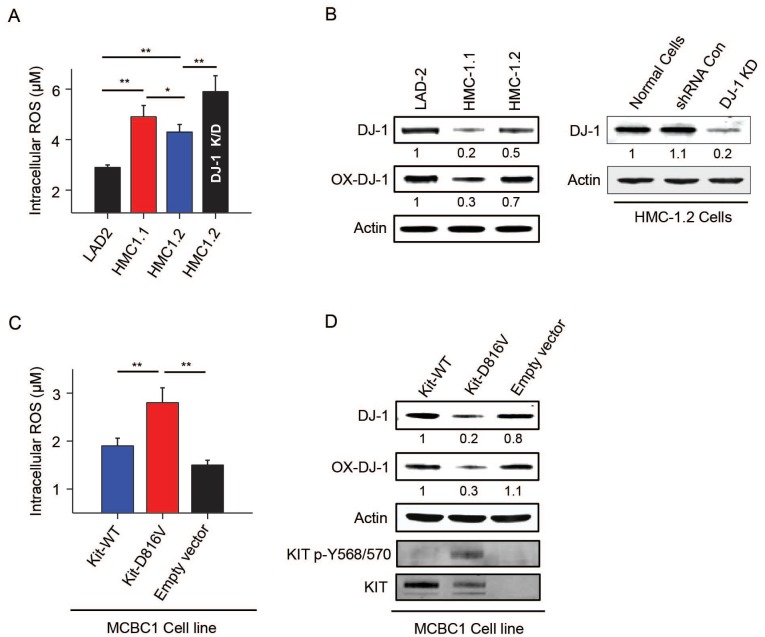
Constitutive KIT activation in cells with gain-of function KIT mutations causes increased ROS levels and decreased DJ-1. (A) Comparison of the intracellular levels of ROS (left panel) and (B) oxidized DJ-1 (Cys 106) and total DJ-1 in human mast cells carrying normal *KIT* (LAD2) or mutated *KIT* (HMC-1.1 and HMC-1.2). DJ-1 expression was silenced in HMC-1.2 using specific sh-RNA sequences to target DJ-1 (DJ-1 KD) or sh-RNA non-target control sequences (sh-RNA Con) (B, right panels). ROS levels in DJ-1 KD and sh-RNA Con cells were measured side by side, but for simplicity, ROS levels in sh-RNA Con are not shown in A since the levels were identical to untreated HMC-1.2 cells (sh-RNA Con: 4.24 μM±0.075 μM). Levels of ROS (C) and DJ-1 (D) in murine MCBS1 mast cells transduced with WT-human *KIT*, D816V-human *KIT* or empty vector as indicated. Presence of KIT and phosphorylated KIT in cells with D816V are shown by western blot (D, bottom panels). The values under the blots indicate average fold increases in the band intensities of DJ-1 or oxidized DJ-1 (corrected to their respective β-actin loading control) as compared to non-stimulated cells (n = 3); SDs were less than 10% of the mean

As gain of function mutations in *KIT* are present in 90% of adult mastocytosis patients and mutations in tyrosine kinase oncogenes can cause ROS accumulation [40], we examined whether mast cells carrying *KIT* mutations have abnormal ROS and DJ-1 levels reminiscent of patients with SM. Because mast cells bearing mutations in KIT from patients with mastocytosis could not be obtained in sufficient numbers, we used mast cell lines with *KIT* mutations derived from a patient with mast cell leukemia, namely HMC-1.1 and HMC-1.2 [32]. The baseline levels of ROS in HMC-1 cells were higher than those in LAD2 mast cells (Fig 2A) which do not have a mutation in *KIT*. Conversely, the levels of DJ-1 were lower in HMC-1 than in LAD2 cells (Fig 2B), suggesting that constitutive activation of KIT results in redox imbalances that may depend in part on DJ-1 depletion. Knockdown of DJ-1 in HMC-1.2 (Fig 2B) and LAD2 cells (data not shown) by shRNA caused increased ROS levels (Fig 2A), while control shRNA did not, demonstrating an antioxidant role for DJ-1 in mast cells. The link between *KIT* mutations and the changes in ROS and DJ-1 was directly demonstrated in a *KIT*-deficient murine mast cell line (MCBS1) [34] which showed increased levels of ROS (Fig 2C) and reduced levels of DJ-1 (Fig 2D) when stably expressing D816V-human KIT in comparison to the same cells expressing normal human-KIT or empty vector.

### KIT regulates ROS production, DJ-1 oxidation, secretion and turnover in human mast cells

**Fig 3:**
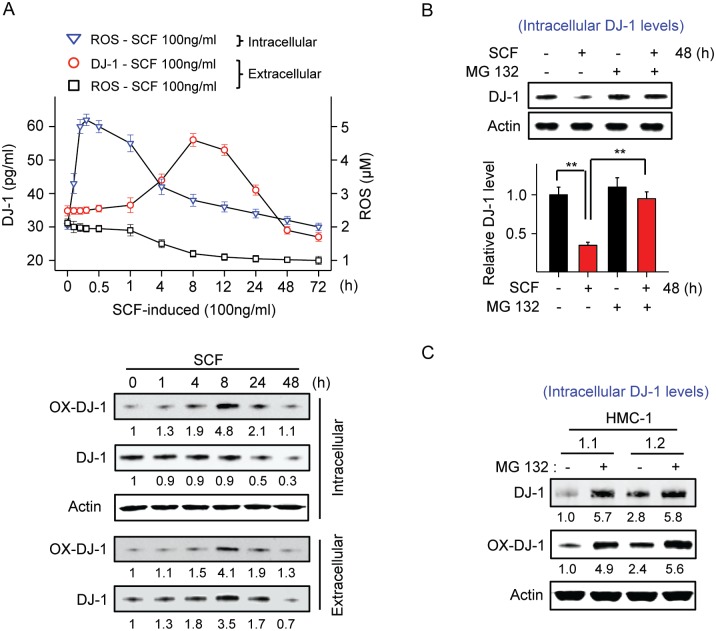
Activation of KIT by SCF induces ROS production and DJ-1 oxidation followed by both DJ-1 release and degradation. (A) Changes in intra- and extracellular ROS and release of DJ-1 into the media (measured by ELISA) in LAD2 cultures after treatment with SCF (100 ng/ml) for the indicated times. The lower panels are immunoblots showing changes in total DJ-1 and oxidized DJ-1 (at Cys 106) inside cells or secreted to the media, as indicated. Shown under the blots are their average relative band intensities (corrected by β-actin intensity) as compared to non-stimulated HMC1.2 cells (n = 3; SDs were less than 10% of the mean). (B) Intracellular DJ-1 levels in LAD2 cells treated with 100 ng/ml SCF for 48 h in the presence or absence of the proteosomal inhibitor MG132 (10 μM) added 6 h prior to SCF stimulation. Intracellular levels of DJ-1 were determined by Western blot. The bar graph shows the average relative band intensities compared to untreated cells (n = 3). (C) Levels of intracellular (total and oxidized) DJ-1 after treatment of HMC-1 cells with the proteosomal inhibitor MG132 (10 μM) for 6 h. All experiments were repeated at least 3 times and data represents mean±SEM. *P<0.*05* and **P<0.*01*.

We then examined the temporal changes in ROS generation following KIT activation in mast cells and the corresponding changes in DJ-1 levels, and its oxidation and secretion into the extracellular media. Stimulation of LAD2 cells with SCF (100 ng/ml) resulted in a rapid and substantial increase in ROS (maximal by 30 min) (Fig 3A) followed by increased oxidation of DJ-1 in Cys 106 within 1–4 h to reach a maximum at 8 h post-activation (Fig 3A, lower panels). The peak in DJ-1 oxidation coincided with the decline in intra- and extracellular ROS levels, consistent with the scavenger role for DJ-1 by self-oxidation, and with DJ-1 secretion into the media (Fig 3A). No changes in the total levels of intracellular DJ-1 were observed within this time period (Fig 3A, lower panels).

Nevertheless, at late times after KIT activation (around 48 h), both oxidized DJ-1 and total DJ-1 significantly declined below baseline levels within and outside the cells (Fig 3A upper and lower panels). This decline was dependent on KIT signals and ROS production, since it was prevented by inhibition of PI3K and ERK1/2, which also inhibited ROS increase and DJ-1 secretion into the media, but not by inhibition of JNK and p38 (S1A–S1E Fig). Treatment with the proteosomal inhibitor MG132 (10 μM) significantly blocked the late reduction of intra- (Fig 3B) and extracellular levels of DJ-1 (S2A Fig) induced by SCF. Our results are consistent with reports that self-oxidation of DJ-1 facilitates both its secretion into the extracellular media and its proteosomal degradation [12, 14, 41–43]. Although a modest reduction (16%) in the transcription of DJ-1 was also apparent by 24 to 48 h after SCF stimulation (S1F Fig), degradation appears to be the major mechanism for the late decline in DJ-1 levels.

We thus next examined whether this increased proteosomal degradation of DJ-1 in LAD 2 cells after prolonged SCF activation is relevant to the reduced levels of DJ-1 in HMC-1.1 and HMC-1.2 given their constitutive KIT activity and increased ROS levels. Treatment of HMC-1 cells with the proteosomal inhibitor MG132 substantially increased oxidized DJ-1 and the baseline intra- (Fig 3C) and extracellular DJ-1 levels (S2B Fig), indicating that cells with constitutive KIT activity, as in LAD2 cells chronically stimulated with SCF (Fig 3B and S2A Fig), have a higher turnover of DJ-1.

### IL-6 regulation of DJ-1 and ROS

**Fig 4:**
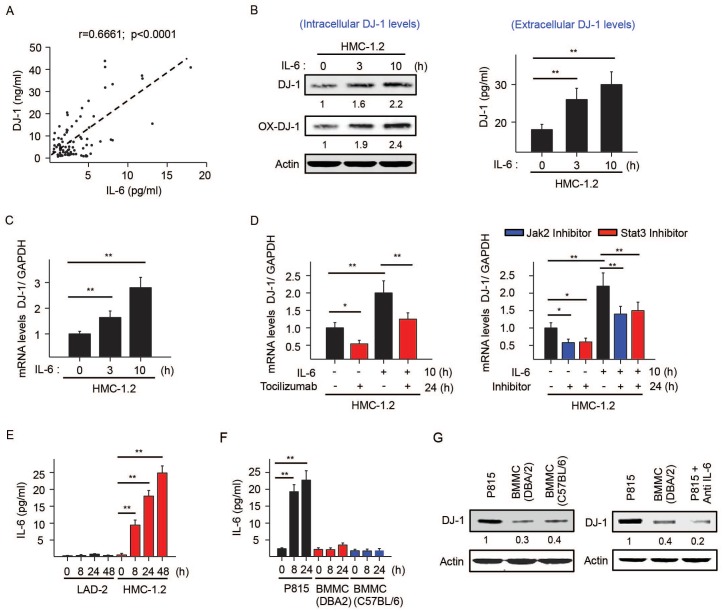
IL-6 is constitutively produced by mast cells carrying D816V-KIT mutations and induces DJ-1 transcription. (A) Positive correlation between DJ-1 and IL-6 serum levels in SM patients. (B) Changes over time in the intracellular levels of total and oxidized DJ-1 (left panel) or secreted DJ-1 (right panel) induced by IL-6 (50 ng/ml) in HMC-1.2. (C) Changes in DJ-1 mRNA expression induced by IL-6 in HMC-1.2 measured by q-RT-PCR. Data are represented as ΔΔCt (compared to unstimulated cells). (D) Effect of the neutralizing IL-6R antibody tocilizumab (50 μg/ml) (left panel), JAK (TG101348; 1 μM) or STAT3 inhibitors (NSC74859; 100 μM) on IL-6 induced DJ-1 transcription. HMC-1.2 cells were pretreated with tocilizumab or the indicated inhibitors for 14 h and incubated with or without 50 ng/ml IL-6 for an additional 10 h, for a total of 24 h. RNA was extracted and DJ-1 transcripts were measured by qRT-PCR. (E) IL-6 secreted into the media by LAD2 and HMC-1.2 cells at the indicated times after replenishing media. Values represent mean±SEM (n = 3). *P<0.05 and **P<0.01. (F) Release of IL-6 into the culture media of P815 or BMMC from the indicated mouse strains. Cells were plated in fresh media in 12 well plates (2 x 10^5^ cells/ well; 1 ml) and supernatants collected after 8 or 24 h. IL-6 was measured by ELISA. (G) Levels of DJ-1 in P815 cells from lysates of cells treated or not with anti-IL6R antibody (100 μg/ml) for 12 h. Shown under the blots are their average relative band intensities (corrected by β-actin intensity) as compared to non-stimulated HMC1.2 cells (n = 3; SDs were less than 10% of the mean).

Although increased turnover of DJ-1 due to activating *KIT* mutations and associated ROS production helps explain the low levels of DJ-1 in ISM patients with relatively low tryptase levels, it was unclear why patients with more aggressive disease had elevated DJ-1 levels. To understand the basis for this unexpected elevation, we examined whether biomarkers known to be associated with high mast cell burden [22, 23, 44–47] could increase DJ-1 expression, thus counteracting the increased turnover of DJ-1 by constitutive KIT activation. IL-6 was of interest because serum IL-6 levels in mastocytosis correlate with tryptase and elevated IL-6 is a better predictor than other cytokines for disease progression [23]. Further, in patients with mastocytosis in our cohort, IL-6 levels significantly correlated with the levels of DJ-1 (Fig 4A). In addition, treatment of HMC1.2 cells with IL-6 increased intra and extracellular DJ-1 protein (Fig 4B). The increased DJ-1 protein levels were found to be a consequence of transcriptional induction of DJ-1 message (Fig 4C) and were abrogated by the neutralizing anti-IL-6R antibody, tocilizumab, as well as JAK and STAT3 inhibitors (Fig 4D), thus demonstrating dependence on IL-6R signaling through JAK/STAT3 pathways. IL-6 induced a similar transcriptional induction of DJ-1 in LAD2 cells pre-stimulated with SCF for 48h (S3A Fig). This was in contrast to two other biomarkers associated with elevated tryptase levels in mastocytosis, IL-31 and histamine [23, 45–47], which had no effect on DJ-1 expression (S3B and S3C Fig).

Tocilizumab and the JAK and STAT3 inhibitors not only prevented IL-6-induced transcription of DJ-1, they also reduced the baseline mRNA levels of DJ-1 in HMC-1.2 cells (Fig 4D), suggesting that IL-6 is produced by HMC-1.2 and acts in an autocrine manner to maintain DJ-1 transcription. In support for this concept, HMC-1.2 cells released substantial amounts of IL-6 into the culture media while LAD2 (Fig 4E) and HMC-1.1 mast cells did not (not shown). Similarly, murine mastocytoma mast cells (P815) carrying a D814Y mutation in *KIT* [48] analogous to the human D816V mutation, constitutively produced high levels of IL-6, while normal BMMC did not (Fig 4F). Furthermore, treatment of P815 cells with a neutralizing anti-IL-6R antibody unmasked reduced DJ-1 expression levels compared to normal BMMCs (Fig 4G), supporting our findings of the opposing influences of IL-6 and chronic KIT activation on DJ-1 in mast cells.

**Fig 5:**
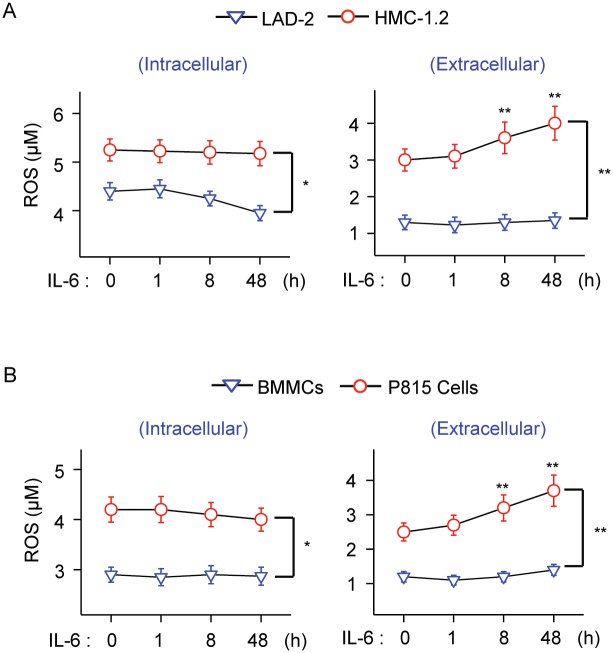
IL-6 increases extracellular ROS levels in HMC-1 cells. (A) Changes in intracellular (left) and extracellular ROS (right) levels induced by IL-6 (50 ng/ml) in HMC-1.2 cells compared to LAD2 cells. LAD2 were pre-stimulated with SCF for 48 h (100 ng/ml) prior to IL-6 stimulation. (B) Changes in intracellular (left) and extracellular ROS (right) levels induced by 50 ng/ml IL-6 in P815 cells compared to normal BMMC. ROS was measured using a fluorescence assay. All experiments were repeated at least 3 times and data represents mean±SEM. *P<0.*05* and **P<0.*01*.

Stimulation with IL-6 did not increase intracellular ROS in mast cells (Fig 5A and 5B, left panels). However, IL-6 did increase the efflux of ROS to the extracellular media in *KIT* (D816V)-mutated HMC-1.2 cells and P815 cells, but not in LAD2 cells or normal BMMC (Fig 5A and 5B, right panels). Unlike for macrophages and neutrophils, activation of mast cells usually results in intracellular accumulation of ROS instead of efflux of ROS to the extracellular space [26]. IL-6, in the cellular context of *KIT* mutations, not only induces DJ-1 expression in mast cells, it promotes secretion of ROS into the extracellular space, a feature that has been linked to some hematological malignancies and to cell transformation in general [1, 49, 50].

### Changes in serum DJ-1 and ROS levels by adoptive transfer of mastocytoma P815 cells into mice and effects of anti-IL-6R

**Fig 6:**
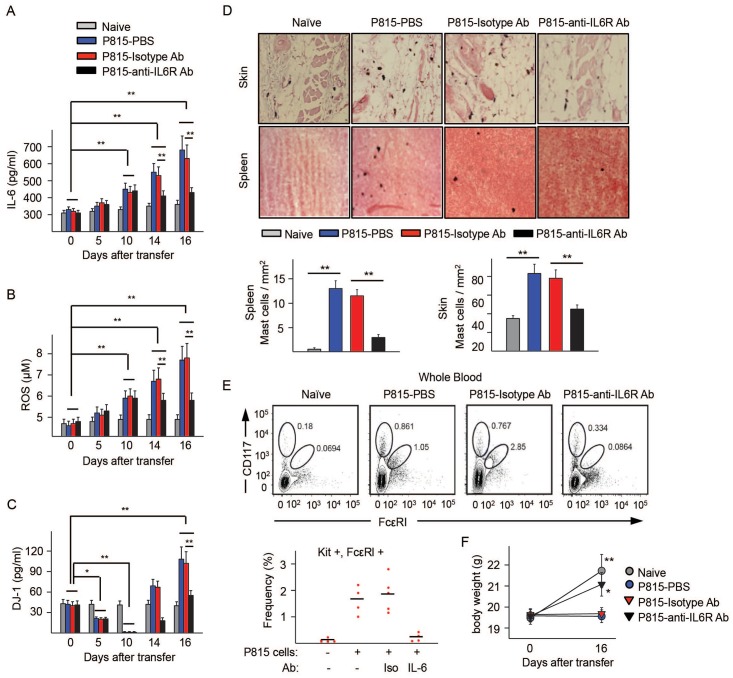
Adoptive transfer of mastocytoma P815 murine cells into DBA/2 mice causes serum alterations in DJ-1 and ROS that are reversed by anti-IL-6R antibody treatment. (A-C) Serum levels of IL-6 (A), ROS (B), and DJ-1 (C) in mice injected with P815 cells and given daily injections of 200 μg/mouse of anti-IL-6R antibody, isotype control antibody or PBS on days 10 through 16 as indicate. (D) Representative histologic images of dorsal skin and spleen samples stained with alcian blue/safranin to detect mast cell in naïve and P185-injected mice untreated or treated with either an isotype control or anti-IL-6R antibody. Below, average number of mast cells per field (5 fields/mouse, n = 4–5) (Axioskop2 plus microscope, total magnification 400X). (E) Representative plots of APC-KIT positive cells (P815 cells lack FcεRI in culture) and APC-KIT and PE-FcεRI double positive cells from blood sorted by FACS in the same experiment. Shown below are the average percentages of mast cells (KIT and FcεRI double positive) in circulation from the various groups of mice. The percentages of KIT positive cells in circulation were as follows: naïve: 0.36±0.07; P815-PBS: 0.91±0.03; P815-Isotype Ab: 0.58±0.03; P815-Anti-IL-6R Ab: 0.21±0.09. (F) Body weights of mice before adoptive transfer of P815 cells and at day 16 after transfer in all the indicated groups. Values represent mean ± SEM of a representative experiment using 4–5 mice per condition. *P<0.05 and **P<0.01. The experiment was repeated twice with similar results.

We next determined whether a murine model of SM induced by injection of P815 cells [31] exhibits the same changes in serum DJ-1 and ROS levels as in human disease. P815 cells, as do HMC1.2 cells, express an activating mutation in *KIT* and have higher levels of ROS than normal BMMC from either DBA/2 or C57/B6 mice (Fig 5B and S4A Fig), and produced high levels of IL-6 in culture (Fig 4F). Adoptive transfer of 2 x10^2^ P815 cells into syngeneic DBA/2 mice, but not of normal BMMCs, resulted in progressive increases in serum IL-6 and ROS that were noticeable by day 10 (Fig 6A and 6B and S4B and S4C Fig). DJ-1 levels, in contrast, showed biphasic changes, dropping to undetectable levels by day 10 compared to naïve mice and dramatically increasing by day 16 (Fig 6C and S4D Fig). The drop in the levels of DJ-1 concomitant with elevated ROS at days 5 to 10 was reminiscent of the low levels of DJ-1 found in ISM patients with lower mast cell burden (Fig 1B). By day 16, when mast cells were found in blood and populated organs such as skin and spleen (Fig 6D and 6E), ROS and DJ-1 levels increased (Fig 6B and 6C) in a similar manner as those in ISM patients with high tryptase or advanced disease. These results demonstrate that alterations in a small population of transformed mastocytoma cells can cause systemic changes in the levels of serum IL-6, ROS and DJ-1 as mast cell burden progresses that resemble those described in Fig 1 for the groups of patients with mastocytosis. Even though this mouse model represents ASM/mast cell leukemia when fully manifested, the accumulation of transformed mast cells is progressive and the associated changes in circulating ROS, DJ-1, and IL-6 at the initial stages parallel the changes observed in ISM.

**Fig 7:**
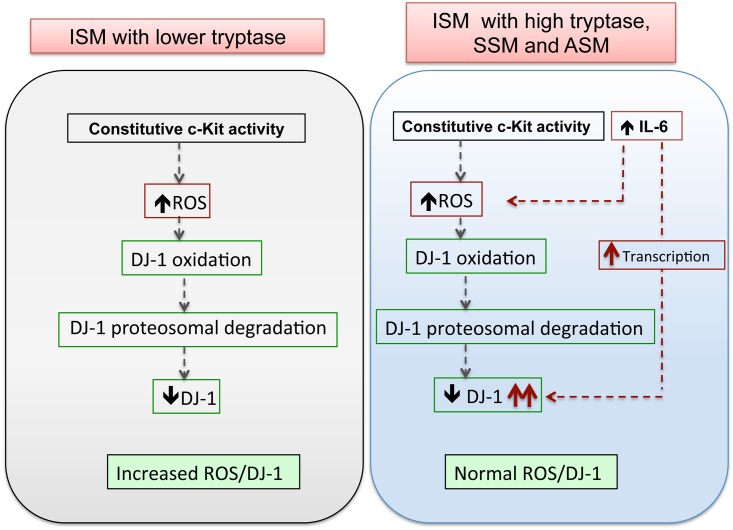
Proposed mechanisms for the alterations in ROS and DJ-1 homeostasis in SM. ISM patients with lower mast cell burden, tryptase values and IL-6 levels show a high ratio of ROS to DJ-1 (>22) as compared to healthy controls (<4) due to their reduced levels of DJ-1. This is driven by constitutive KIT activity in *KIT* mutated MCs which induces ROS production and causes oxidation and proteosomal degradation of oxidized DJ-1 (left panel). In ISM patients with higher mast cell burden and with advanced disease, the increasing levels of DJ-1 offset the ROS to DJ-1 ratio toward normal values (<8; see Fig 1C). This is driven by elevated levels of IL-6 in these patients, which causes DJ-1 transcription and compensates for the enhanced DJ-1 turnover. Reduction of oxidative imbalances may allow for MC expansion.

To test whether progressive increases in IL-6 are responsible for the rise in ROS and DJ-1 *in vivo*, as was demonstrated *in vitro* (Figs 4 and 5), we blocked IL-6R signaling by sequential injections of anti-IL-6R antibody. Anti-IL-6R antibody injected 10 days after the transfer of P815 cells, when serum IL-6 began to increase significantly, prevented further increases in IL-6 (Fig 6A) and significantly inhibited the substantial rises in ROS (Fig 6B) and DJ-1 (Fig 6C) by day 16, while injection of an isotype control antibody had no effect. Of importance, treatment of P815-adoptively transferred mice with anti-IL6 receptor antibody markedly reduced the increase in numbers of mast cells in tissues (Fig 6D) and blood (Fig 6E). Interestingly, anti-IL-6 antibody-treated mice, unlike mock-treated mice, gained nearly as much weight as naïve mice, indicating a healthier overall condition (Fig 6F). Thus, IL-6 in the mouse model, as in mast cell cultures, mediates the increases in circulating DJ-1 and ROS observed after day 10 and supports the concept of an involvement of IL-6 in the changes in redox regulation in SM patients with greater mast cell burden (Fig 7). The data also demonstrate that blocking IL-6 signaling hinders mast cell expansion and, of possible therapeutic interest, suggest that IL-6-induced changes in DJ-1 and ROS may promote mast cell expansion.

Unedited, full size blots of Figs 2–4 are all shown in S6 Fig.

## Discussion

Increased ROS and oxidative stress occurs frequently in malignancies and myeloproliferative diseases [1, 2, 40, 49]. DJ-1, a multifunctional protein with both antioxidant function and cytoprotective activity against oxidative stress, increases with cancer progression [7, 8], although the mode of DJ-1 regulation has not been studied in detail. Here, we investigated the regulation of DJ-1 in relationship with ROS and mastocytosis severity. DJ-1 levels were reduced in ISM with low mast cell burden but, in marked contrast, increased in patients with high mast cell burden or with advanced disease. Our findings in mast cells carrying *KIT* mutations and in a mouse model of mastocytosis suggest a biphasic regulation in which constitutive KIT activation drives continuous DJ-1 oxidation and degradation while escalated IL-6 production in more severe mastocytosis variants induces DJ-1 transcription (see model in Fig 7). We propose that induction of DJ-1 by IL-6 and reduction of the ratio ROS to DJ-1 contribute to mast cell expansion in more severe disease. The effectiveness of IL-6 blockade, minimizing ROS and DJ-1 levels and preventing the progressive accumulation of mast cells in the mouse model, suggest that administration of anti-IL-6R, inhibition of IL-6-triggered signaling or both may be considered as an adjunctive therapeutic approach for aggressive mastocytosis.

Of relevance to the elevated serum ROS levels in patients with mastocytosis, we find that gain-of-function mutations in *KIT* cause overproduction of ROS in human mast cells. Similarly, mutations in other tyrosine kinase receptors commonly associated with myeloproliferative diseases such as BCR/ABL or FLT3ITD cause increased ROS in myeloid cells [40, 51]. The increase in ROS in mast cell lines derived from mastocytosis patients and in ISM with a low mast cell burden was accompanied by low DJ-1 levels. Depletion of DJ-1 or other protective antioxidant molecules is also found in conditions that cause chronic exposure to oxidative stress such as cigarette smoke [41] and malignancies [1, 52]. Our studies in human mast cells show that activation of KIT with its endogenous ligand, SCF, induces intracellular generation of ROS similar to that induced by antigen [25, 26, 53], followed by the oxidative modification of DJ-1 which, consistent with previous reports [14, 41–43, 54, 55], facilitates DJ-1 export out of cells as well as its degradation, resulting in a long-term decline in DJ-1 levels. This decline is dependent on ERK1/2 and PI3K signals. ROS-induced oxidation, export, and turnover of DJ-1 leads to depletion of DJ-1 pools and may thus explain the reduction in serum DJ-1 levels in patients with ISM with low mast cell burden. Such a scenario is supported by our finding that adoptive transfer of *KIT*-mutated mast cells into mice remarkably reproduced the changes in serum DJ-1 (decline and later increase) and ROS observed in patients with SM as the mast cell burden and disease severity increases.

An unexpected finding was the shift in pattern of DJ-1 regulation as mast cells accumulate in which DJ-1 levels were elevated in patients with advanced mastocytosis as was the case in the mouse model at later times after adoptive transfer of *KIT*-mutated mast cells. We present evidence that the IL-6/JAK/STAT3 pathway mediates this elevation by inducing DJ-1 transcription in cells with *KIT* mutations. A unique feature of DJ-1 among other antioxidants, in addition to its scavenger role, is its control of cellular metabolism [56] and the transcription and stability of molecules that regulate apoptosis and survival, thus conferring cellular protection against oxidative stress [7, 57, 58]. These considerations, together with our findings that knockdown of DJ-1 by shRNA in HMC-1 cells (S5 Fig) or by blockage of IL-6 in the mastocytosis model (Fig 6) reduces mast cell growth in culture or *in vivo*, respectively, suggest that while depletion of DJ-1 in ISM may result in cytostatic effects under oxidative stress, the IL-6-induced restoration of DJ-1 in more aggressive SM may prevent further oxidative damage, favoring mast cell survival and expansion. In agreement, expression of apoptosis genes is increased in mast cells from patients with ISM compared to mast cells from ASM, which instead exhibit enhanced expression of cell cycle genes [59]. Thus, DJ-1 levels or the ratio DJ-1/ROS could be further explored as potential biomarkers of disease severity.

In addition to inducing DJ-1 expression, IL-6 elicited significant release of ROS to the extracellular space but only in transformed mast cells harboring D816V-*KIT* mutations. In non-phagocytic cells, increased efflux of ROS concomitant with increased expression of antioxidant enzymes is associated with cellular transformation [1, 49]. From this perspective, our data are consistent with the reported view of IL-6 over-production as a key factor favoring progression of mastocytosis into more advanced disease [23]. Since IL-6 frequently associates with various tumors [60, 61], transcriptional induction of DJ-1 by IL-6 may represent a more general adaptation mechanism for DJ-1 up-regulation during transformation as a defense against oxidative stress.

Redox adaptation mechanisms in malignant cells not only protect transformed cells from increased ROS, they also result in a decrease in apoptotic execution, elevated DNA repair capability and drug resistance [2, 4]. Due to this dependence on antioxidants for malignant cell survival, a proposed therapeutic strategy has been to abrogate key antioxidant adaptive mechanisms in order to selectively kill these cells with minimal damage to normal cells [2, 4, 5]. DJ-1, elevated in cancers with poor prognosis and in advanced mastocytosis (shown here), has been implicated in cell-resistance to stress and anticancer drugs and thus it represents a potential target to weaken malignant cell advantage [10, 62, 63]. Thus, our study provides a basis for further investigation on therapeutic approaches that target DJ-1 in ASM, a disease for which there is currently no effective treatments. Our animal model provides indirect evidence (i.e. through blocking of IL-6R) of the potential interest of such a strategy.

In summary, we describe for the first time factors regulating DJ-1, in particular the induction of DJ-1 transcription by IL-6 in the context of constitutive KIT tyrosine kinase activation, which may compensate for excessive ROS. These findings provide clues for a better understanding of oxidative stress regulation in mastocytosis, and potentially other neoplastic diseases with *KIT* mutations; as well as the contributions of DJ-1 to this process. As such, activating *KIT* mutations lead to diminished DJ-1 along with oxidative stress; whereas in patients with higher mast cell burdens, IL-6 over-production induces DJ-1 expression, likely conferring protection against toxic levels of ROS and favoring mast cell growth. Our observations support the concept that therapeutic approaches that block DJ-1 transcription, including blockade of IL-6R or its signaling, may be of potential benefit in advanced mastocytosis by reducing mast cell growth at least partly through interfering with redox adaptation mechanisms.

## Supporting Information

S1 Fig Effect of PI3K, ERK1/2, JNK and p38 inhibitors on SCF-induced changes in intracellular ROS and DJ-1 secretion and effect of SCF on the transcription of DJ-1. (A-E) Inhibitors of PI3K (LY294002; 10 μM in A and wortmannin; 100 nM in B), ERK1/2 (U0126; 10 μM in C), JNK (SP600125; 10 μM in D) and p38 (SB203580; 10 μM in E) were added 30 min prior to SCF stimulation (100 ng/ml) in LAD2 cells for the indicated times. ROS in cells and DJ-1 in the media were measured as explained in methods. (F) Changes in DJ-1 mRNA expression induced by 100 ng/ml SCF at the indicated times were measured by qRT-PCR. The relative mRNA DJ-1 levels are expressed as ΔΔCt using GAPDH as control. Data represents mean±SEM (n ≥3). *P<0.*05*.
(DOCX)

S2 Fig Effect of inhibition the proteasome on extracellular DJ-1 in LAD2 and HMC-1 cells. (A) Extracellular DJ-1 levels in LAD2 cells treated with 100 ng/ml SCF for 48 h in the presence or absence of the proteosomal inhibitor MG132 (10 μM) added 6 h prior to SCF stimulation. (B) Extracellular levels of secreted DJ-1 after treatment of HMC-1 cells with the proteosomal inhibitor MG132 for 6 h. DJ-1 levels in the extracellular media were determined by ELISA. All experiments were repeated at least 3 times and data represents mean±SEM. *P<0.*05* and **P<0.*01*.
(DOCX)

S3 Fig IL-6, but not IL-31 or Histamine, induces DJ-1 transcription and increases DJ-1 levels in SCF-stimulated LAD2 cells. Levels of DJ-1 and oxidized DJ-1 (left panels), secreted DJ-1 (middle panels) and DJ-1 mRNA expression (right panels) in LAD2 cells pre-stimulated with SCF for 48 h (100 ng/ml) and then treated with 50 ng/ml IL-6 (A), 50 ng/ml IL-31 (B) or 1 μM histamine (C) for the indicated times. Shown in A, right panel, are the blocking effects of anti-IL-6 receptor antibody tocilizumab (10 μg/ml) on IL-6-induced DJ-1 mRNA expression. Tocilizumab was added 2 h prior to IL-6 stimulation. The values under the blots indicate fold increases in the band intensities of DJ-1 or oxidized DJ-1 (corrected for β-actin loading controls) as compared to non-stimulated cells and represent the mean of three independent experiments. Changes in DJ-1 mRNA expression (right panels) were measured by q-RT-PCR. Data is represented as ΔΔCt (as compared to non-stimulated cells). Experiments were repeated at least 3 times and values represent mean±SEM. *P<0.*05* and **P<0.*01*.
(DOCX)

S4 Fig Adoptive transfer of P185, but not BMMC, leads to increases in mast cell numbers, serum IL-6, ROS and DJ-1. (A) Increased levels of ROS in P815 cells. Intracellular and extracellular levels of ROS in P815 mastocytoma murine cells compared to normal bone marrow mast cells (BMMC) from DBA/2 or C57BL/6 mice. P815 or BMMC from the indicated mouse strains were plated in 12 well plates (2x10^5^ cells/ well; 1 ml) and 8 h later, intracellular ROS content or in the media were measured. Serum levels of IL-6 (B), ROS (C) and DJ-1 (D) at 0, 5, 10 and 16 days after adoptive transfer of 10^2^ P815 mastocytoma cells (black bars), BMMCs (red bars) into mice and by comparison, in naïve DBA mice (grey bars).
(DOCX)

S5 Fig Knockdown of DJ-1 in HMC-1.2 reduces cell growth. HMC-1.2 MCs were transduced with sh-RNA sequences for DJ-1 (DJ-1 KD) or sh-RNA non-target control sequences (sh-RNA Con) as described in the supplementary methods. After selection, 3.0x10^5^ cells were plated in fresh media and their growth within 6 days assessed by counting viable cells.
(DOCX)

S6 Fig Unedited full size blots. Full size blots from Fig 2B and 2D are shown in (A), those from Fig 3A–3C are shown in (B) and full size blots from Fig 4B and 4G in (C). In A-C, quantification of the bands shown in these figures is represented in bar graphs. Boxes in red indicate the lanes shown in the manuscript Figs.
(DOCX)

## Abbreviations

ASM: Aggressive systemic mastocytosis
BMMCs: Bone marrow mast cells
KIT: Stem cell factor receptor
ISM: Indolent systemic mastocytosis
SCF: Stem cell factor
IL-6: Interleukin 6
IL-6R: Interleukin 6 receptor
IL-31: Interleukin 31
JAK: Janus Kinase
PARK7: Parkinson protein 7 (DJ-1)
ROS: Reactive oxygen species
STAT3: Signal transducer and activator of transcription 3
SM: Systemic mastocytosis
SSM: Smoldering systemic mastocytosis
