# Supplementary material for: Regulation of Reactive Oxygen Species and the Antioxidant Protein DJ-1 in Mastocytosis
Source: PLoS One. 2016 Sep 9;11(9):e0162831. doi: 10.1371/journal.pone.0162831 (PMC5017616; doi:10.1371/journal.pone.0162831)
Supplement: S1 Fig — (A-E) Inhibitors of PI3K (LY294002; 10 μM in A and wortmannin; 100 nM in B), ERK1/2 (U0126; 10 μM in C), JNK (SP600125; 10 μM in D) and p38 (SB203580; 10 μM in E) were added 30 min prior to SCF stimulation (100 ng/ml) in LAD2 cells for the indicated times. ROS in cells and DJ-1 in the media were measured as explained in methods. (F) Changes in DJ-1 mRNA expression induced by 100 ng/ml SCF at the indicated times were measured by qRT-PCR. The relative mRNA DJ-1 levels are expressed as ΔΔCt using GAPDH as control. Data represents mean±SEM (n ≥3). *P<0.05. (DOCX) [file pone.0162831.s001.docx]

**S1 Fig- Effect of PI3K, ERK1/2, JNK and p38 inhibitors on SCF-induced changes in intracellular ROS and DJ-1 secretion and effect of SCF on the transcription of DJ-1**
