# Supplementary material for: Regulation of Reactive Oxygen Species and the Antioxidant Protein DJ-1 in Mastocytosis
Source: PLoS One. 2016 Sep 9;11(9):e0162831. doi: 10.1371/journal.pone.0162831 (PMC5017616; doi:10.1371/journal.pone.0162831)
Supplement: S2 Fig — (A) Extracellular DJ-1 levels in LAD2 cells treated with 100 ng/ml SCF for 48 h in the presence or absence of the proteosomal inhibitor MG132 (10 μM) added 6 h prior to SCF stimulation. (B) Extracellular levels of secreted DJ-1 after treatment of HMC-1 cells with the proteosomal inhibitor MG132 for 6 h. DJ-1 levels in the extracellular media were determined by ELISA. All experiments were repeated at least 3 times and data represents mean±SEM. *P<0.05 and **P<0.01. (DOCX) [file pone.0162831.s002.docx]

**S2 Fig. Effect of inhibition the proteasome on extracellular DJ-1 in LAD2 and HMC-1 cells**
