# Supplementary material for: Regulation of Reactive Oxygen Species and the Antioxidant Protein DJ-1 in Mastocytosis
Source: PLoS One. 2016 Sep 9;11(9):e0162831. doi: 10.1371/journal.pone.0162831 (PMC5017616; doi:10.1371/journal.pone.0162831)
Supplement: S3 Fig — Levels of DJ-1 and oxidized DJ-1 (left panels), secreted DJ-1 (middle panels) and DJ-1 mRNA expression (right panels) in LAD2 cells pre-stimulated with SCF for 48 h (100 ng/ml) and then treated with 50 ng/ml IL-6 (A), 50 ng/ml IL-31 (B) or 1 μM histamine (C) for the indicated times. Shown in A, right panel, are the blocking effects of anti-IL-6 receptor antibody tocilizumab (10 μg/ml) on IL-6-induced DJ-1 mRNA expression. Tocilizumab was added 2 h prior to IL-6 stimulation. The values under the blots indicate fold increases in the band intensities of DJ-1 or oxidized DJ-1 (corrected for β-actin loading controls) as compared to non-stimulated cells and represent the mean of three independent experiments. Changes in DJ-1 mRNA expression (right panels) were measured by q-RT-PCR. Data is represented as ΔΔCt (as compared to non-stimulated cells). Experiments were repeated at least 3 times and values represent mean±SEM. *P<0.05 and **P<0.01. (DOCX) [file pone.0162831.s003.docx]

**S3 Fig- IL-6, but not IL-31 or Histamine, induces DJ-1 transcription and increases DJ-1 levels in SCF-stimulated LAD2 cells**
