# Supplementary material for: Regulation of Reactive Oxygen Species and the Antioxidant Protein DJ-1 in Mastocytosis
Source: PLoS One. 2016 Sep 9;11(9):e0162831. doi: 10.1371/journal.pone.0162831 (PMC5017616; doi:10.1371/journal.pone.0162831)
Supplement: S4 Fig — (A) Increased levels of ROS in P815 cells. Intracellular and extracellular levels of ROS in P815 mastocytoma murine cells compared to normal bone marrow mast cells (BMMC) from DBA/2 or C57BL/6 mice. P815 or BMMC from the indicated mouse strains were plated in 12 well plates (2x10^5^ cells/ well; 1 ml) and 8 h later, intracellular ROS content or in the media were measured. Serum levels of IL-6 (B), ROS (C) and DJ-1 (D) at 0, 5, 10 and 16 days after adoptive transfer of 102 P815 mastocytoma cells (black bars), BMMCs (red bars) into mice and by comparison, in naïve DBA mice (grey bars). (DOCX) [file pone.0162831.s004.docx]

**S4 Fig- Adoptive transfer of P185, but not BMMC, leads to increases in mast cell numbers, serum IL-6, ROS and DJ-1**
