# Supplementary material for: Regulation of Reactive Oxygen Species and the Antioxidant Protein DJ-1 in Mastocytosis
Source: PLoS One. 2016 Sep 9;11(9):e0162831. doi: 10.1371/journal.pone.0162831 (PMC5017616; doi:10.1371/journal.pone.0162831)
Supplement: S5 Fig — HMC-1.2 MCs were transduced with sh-RNA sequences for DJ-1 (DJ-1 KD) or sh-RNA non-target control sequences (sh-RNA Con) as described in the supplementary methods. After selection, 3.0x10^5^ cells were plated in fresh media and their growth within 6 days assessed by counting viable cells. (DOCX) [file pone.0162831.s005.docx]

**S5 Fig- Knockdown of DJ-1 in HMC-1.2 reduces cell growth**
