# Supplementary figures and images for: Regulation of Reactive Oxygen Species and the Antioxidant Protein DJ-1 in Mastocytosis
Source: PLoS One. 2016 Sep 9;11(9):e0162831. doi: 10.1371/journal.pone.0162831 (PMC5017616; doi:10.1371/journal.pone.0162831)

**S6 Fig- Unedited full size blots**


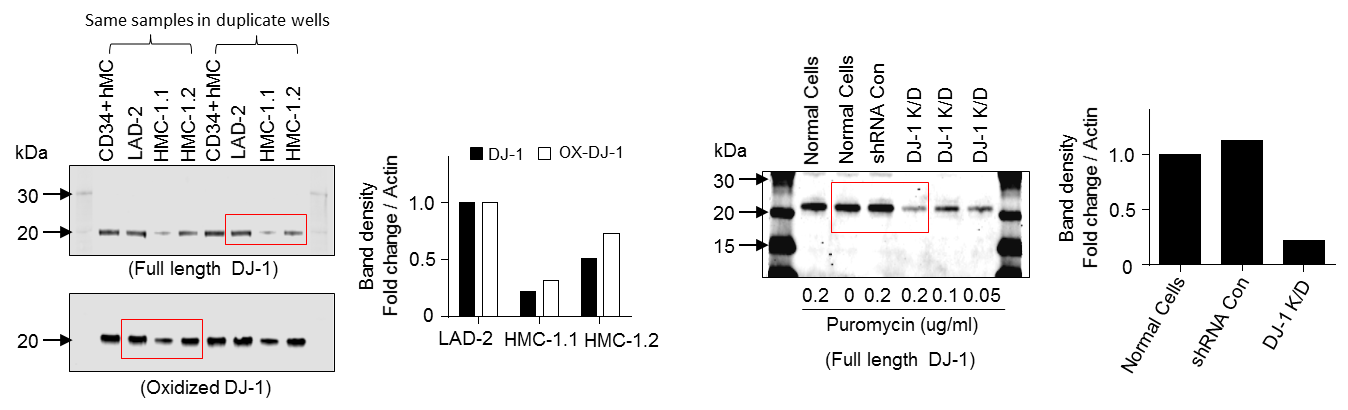
**(Fig 2B)**


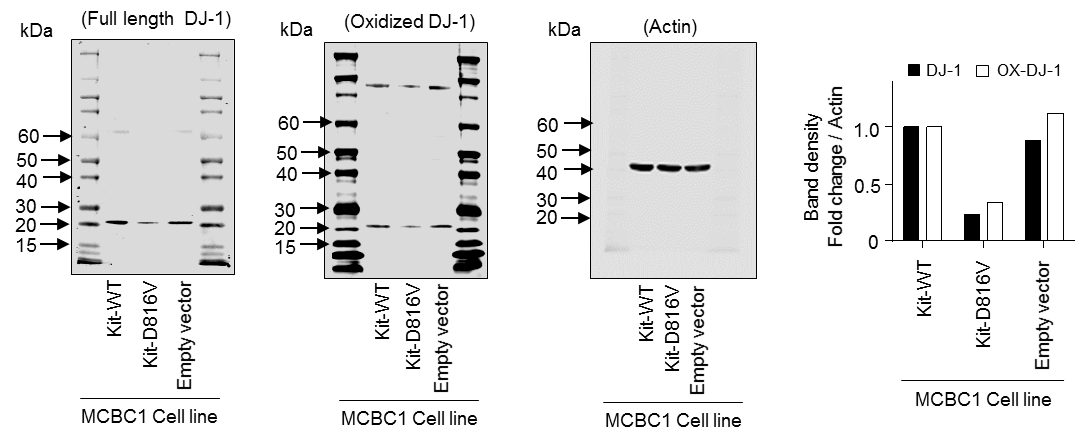
**(Fig 2D)**


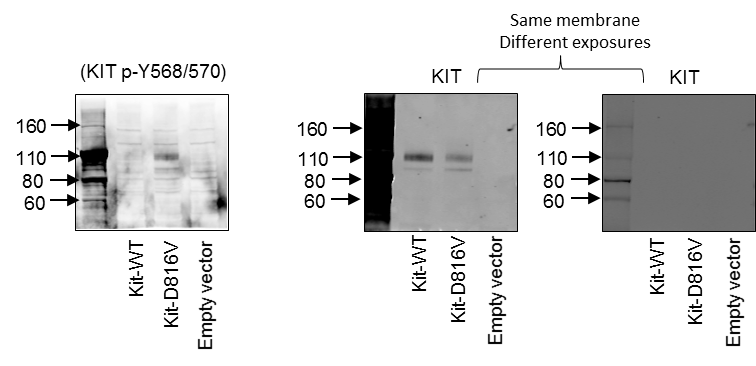


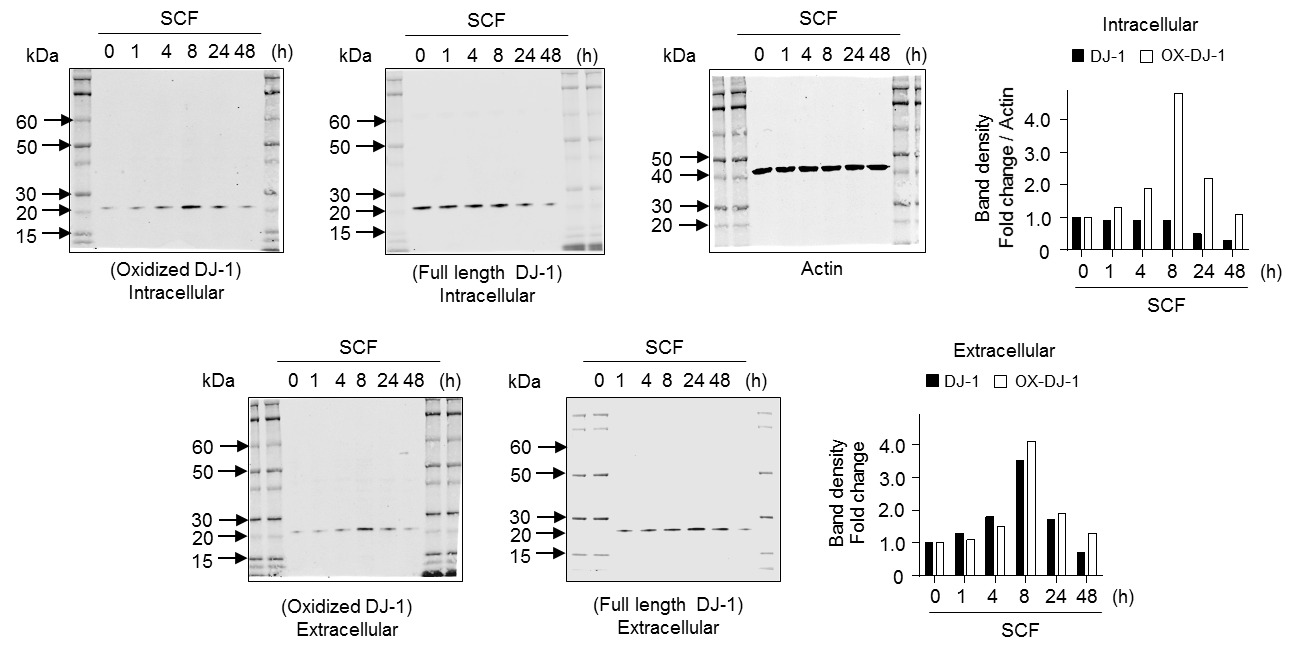
**(Fig 3A)**

**(Fig 3B)**


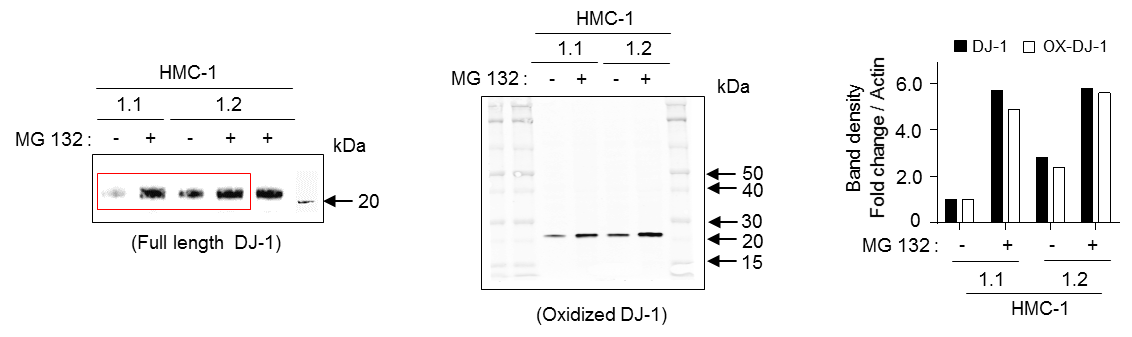

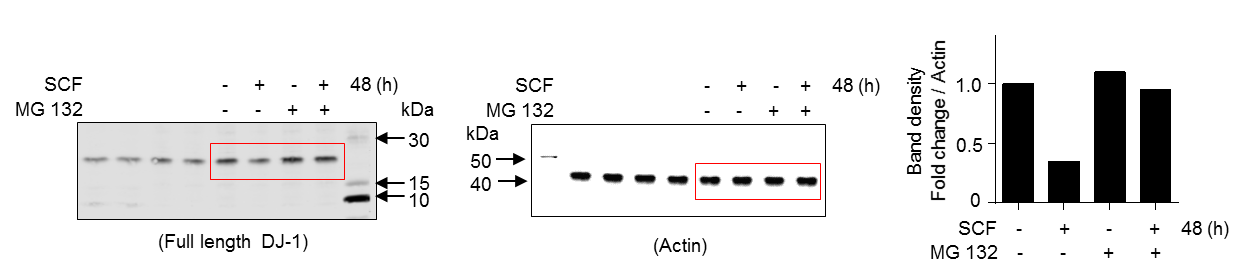


**(Fig 3C)**

**(Fig 4B)**

**
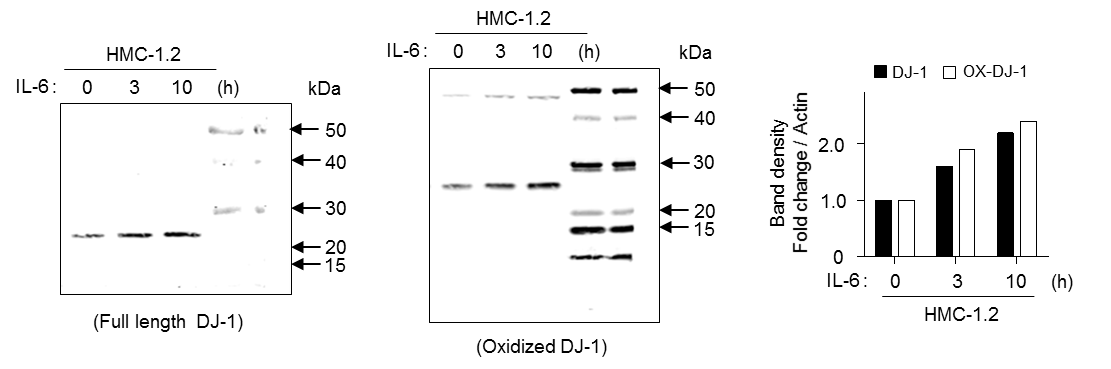
**

**(Fig 4G)**

**
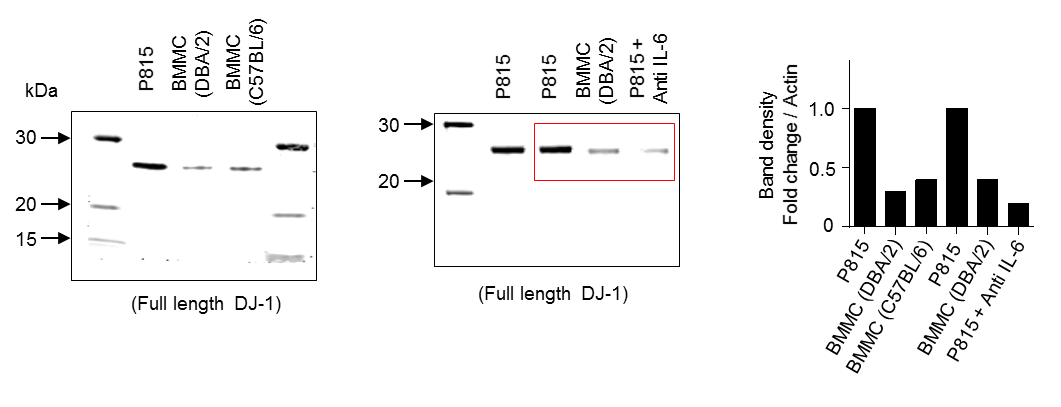
**

Supplement: S6 Fig — Full size blots from Fig 2B and 2D are shown in (A), those from Fig 3A–3C are shown in (B) and full size blots from Fig 4B and 4G in (C). In A-C, quantification of the bands shown in these figures is represented in bar graphs. Boxes in red indicate the lanes shown in the manuscript Figs. (DOCX) [file pone.0162831.s006.docx]
